# Identification and Validation of NAC Transcription Factors Enhancing Phenolic Acid Production in *Perilla frutescens*

**DOI:** 10.3390/plants15060922

**Published:** 2026-03-17

**Authors:** Jiayi Xu, Ping Wang, Junmei Lian, Linqiang Zhang, Xiaobi Zhang, Yan Sui, Jiankang Chen, Heng Wei, Yihan Wang, Rongde Cui, Wanying Li, Nanqi Zhang, Yan Yan, Jian Zhang, Peng Di

**Affiliations:** 1 State Local Joint Engineering Research Center of Ginseng Breeding and Application, College of Chinese Medicinal Materials, Jilin Agricultural University, Changchun 130118, China; 2 Department of Biology, University of British Columbia, Okanagan, Kelowna, BC V1V 1V7, Canada; 3 Faculty of Agronomy, Jilin Agricultural University, Changchun 130118, China

**Keywords:** *Perilla frutescens* (L.) Britt, NAC transcription factor family, hairy roots, phenolic acids

## Abstract

Phenolic acids are the major bioactive compounds in *Perilla frutescens* (L.) Britt; however, the regulatory roles of NAC transcription factors (TFs) in their biosynthesis remain unclear. Here, we performed a genome-wide identification and characterization of the NAC family in *P. frutescens* and explored their involvement in phenolic acid production. A total of 108 PfNAC genes were identified and classified into 17 subfamilies. Expression and promoter analyses suggested potential roles in secondary metabolism. *PfNAC29* is located in the plasma membrane and necleus, while *PfNAC40* and *PfNAC80* are located in the nucleus.Yeast one-hybrid and dual-luciferase assays demonstrated that these TFs bind to the CATGTG motif in the *PfC4H* promoter and activate its transcription. Overexpression in transgenic hairy roots significantly increased rosmarinic acid, caffeic acid, and ferulic acid accumulation, accompanied by upregulation of key biosynthetic genes. These results indicate that *PfNAC29*, *PfNAC40*, and *PfNAC80* act as positive regulators of phenolic acid biosynthesis and provide promising targets for metabolic engineering in medicinal plants.

## 1. Introduction

Transcription factors (TFs) are proteins with specific structural features that play essential roles in regulating plant growth and development [1]. The NAC (NAM, ATAF1/2, and CUC) transcription factor family is one of the largest TF families in plants and is characterized by its distinct structural organization. The N-terminal region of NAC proteins contains a highly conserved DNA-binding domain of approximately 150 amino acids, which is crucial for recognizing and binding to specific DNA sequences. In contrast, the C-terminal region is a transcriptional regulatory domain that is highly variable, conferring functional diversity and flexibility in gene regulation. The NAC DNA-binding domain can be further divided into five subdomains (A–E). Among them, subdomains C and D are highly conserved and contain nuclear localization signals, which are likely involved in promoter recognition and specific DNA interactions. Subdomain A is also conserved across species and may contribute to NAC protein dimerization, while subdomain E, though less conserved, can cooperate with subdomain D in DNA binding and may be involved in developmental or tissue-specific regulation.

The NAC family was first identified in *Petunia hybrida* [2]. With the advancement of genome sequencing technologies, genome- and transcriptome-wide identification of NAC family genes has become possible in various plant species. To date, a total of 98 PnNAC genes have been identified in *Panax notoginseng* [3], 130 NAC genes in sweet cherry (*Prunus avium*) [4], and 109 NAC genes in *Madhuca longifolia* [5]. These findings suggest that the NAC transcription factor family is widely distributed among plant species and has undergone remarkable evolutionary expansion. Numerous studies have demonstrated that NAC proteins perform diverse and essential functions in plants, such as regulating developmental processes and delaying or suppressing leaf senescence [6], promotion of lateral root development [7], and regulation of responses to biotic and abiotic stresses [8,9]. In secondary metabolism, several NAC proteins have been reported to participate in phenolic acid biosynthesis. For example, *SmNAC36* activates the expression of *SmMYB18*, which in turn upregulates *SmTAT2*, thereby promoting the accumulation of phenolic acids in *Salvia miltiorrhiza* [10]. Overexpression of *SmNAC1* was also found to alter the accumulation of salvianolic acids (SalA) by regulating key biosynthetic enzymes in *S. miltiorrhiza* [11]. In *Perilla frutescens* (L.) Britt, the light-responsive factor *PfGBF3* positively regulates *PfNAC2* and *PfC4H* to enhance rosmarinic acid (RA) production, while *PfNAC2* itself directly activates *PfC4H* expression under high light [12]. To date, the NAC transcription factor family in *P. frutescens* has not been systematically identified or functionally characterized.

*P. frutescens* is an important medicinal and edible plant widely cultivated in East Asia. It is rich in phenolic compounds, which have gained significant attention in recent years. Phenolic acids, the major water-soluble secondary metabolites in *P. frutescens*, are characterized by a polyhydroxyphenylpropanoid structure and exhibit a wide range of biological activities, including anti-allergic properties [13], antitumor effects [14], developmental regulation [15], and plant growth regulation [16]. With the rapid growth of the global health-related industries and increasing demand for natural bioactive compounds, the cultivation scale and market value of *P. frutescens* have risen significantly, establishing it as an important raw material for high-value natural products. In *P. frutescens*, phenolic acids are primarily synthesized through the phenylpropanoid and tyrosine-derived pathways [17]. Recent studies have identified several transcription factors involved in the regulation of these pathways. For instance, *PfbHLH13* promotes phenolic acid biosynthesis by activating key genes in the phenylpropanoid pathway, while *PfbHLH66* simultaneously regulates both metabolic routes [18]. However, current research remains fragmented, with most studies focusing on individual genes or localized regulatory mechanisms, while systematic studies on the metabolic network are still lacking. Specifically, although the NAC transcription factor family is known to play critical roles in plant development, stress responses, and secondary metabolism, its regulatory function in phenolic acid biosynthesis in *P. frutescens* remains poorly understood, and no comprehensive, genome-wide study has been conducted. Therefore, a genome-wide identification and functional characterization of the NAC transcription factor family in *P. frutescens* is crucial for understanding the transcriptional regulatory network underlying phenolic acid metabolism. This research will not only deepen our knowledge of the regulatory mechanisms in this important medicinal plant but also provide valuable targets for molecular breeding and metabolic engineering to enhance the production of phenolic acid-derived natural products with high pharmaceutical and economic value.

To address the existing knowledge gap, a comprehensive genome-wide analysis of the NAC transcription factor family in *P. frutescens* was conducted based on the latest genomic data [19]. A total of 108 PfNAC transcription factors were identified, with an uneven distribution across 20 chromosomes. Their conserved motifs, gene structures, cis-regulatory elements, and phylogenetic relationships were systematically analyzed. Among these, three representative genes—*PfNAC29*, *PfNAC40*, and *PfNAC80*—were selected for functional validation due to their high homology with *PfNAC2* (KAH6818974.1). Subcellular localization, yeast one-hybrid assays, and dual-luciferase reporter assays indicated that these PfNACs likely bind to the CATGTG motif in *PfC4H* and function as transcriptional activators. Further analysis revealed that overexpression of *PfNAC29*, *PfNAC40*, and *PfNAC80* in transgenic hairy roots significantly enhanced phenolic acid accumulation, confirming their role as positive regulators. Collectively, this study provides new insights into the structural and functional diversity of the PfNAC family and reveals the pivotal regulatory roles of PfNACs in phenolic acid biosynthesis. These findings offer a theoretical foundation for molecular breeding and metabolic engineering of *P. frutescens*, aiming to enhance the production of bioactive phenolic compounds.

## 2. Results

### 2.1. Chromosomal Distribution and Characteristics of PfNAC Genes

**Figure 1:**
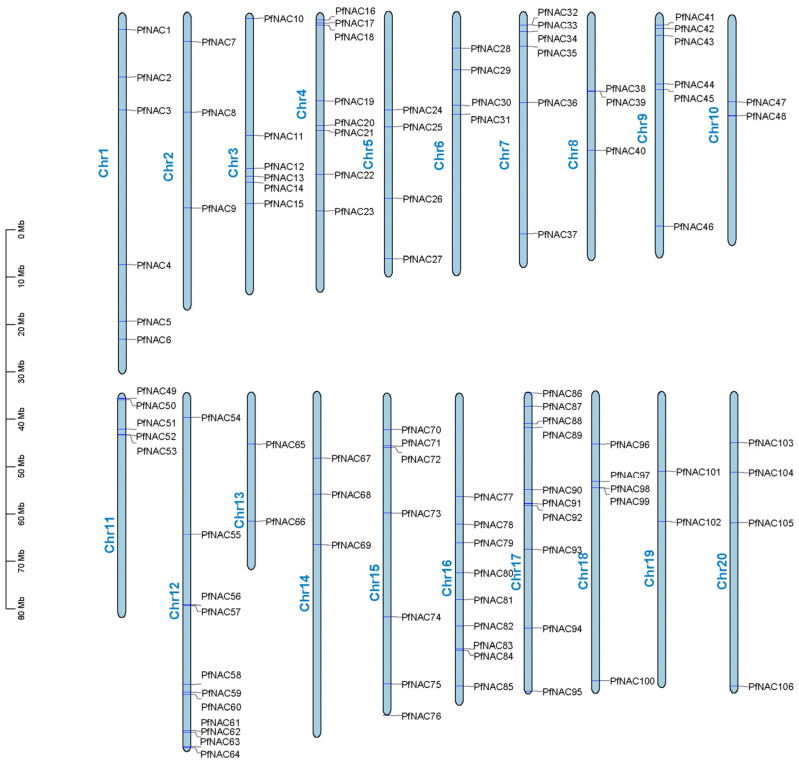
Chromosomal distribution of PfNAC genes. Chromosome numbers are indicated in blue.

Based on a genome-wide search of NAC sequences in *P. frutescens*, a total of 108 PfNAC transcription factors were identified (Supplementary Table S1). Chromosomal localization analysis using TBtools showed that 106 PfNAC genes were unevenly distributed across 20 chromosomes (Figure 1), with the number of genes per chromosome ranging from 2 to 11. Chromosomes 12 and 17 contained the largest numbers of PfNAC genes (11 and 10, respectively), whereas chromosomes 10, 13, and 19 each harbored only two genes. Most PfNAC genes on chromosomes 8, 10, and 11 were mainly located in the central chromosomal regions. Two PfNAC genes were not assigned to any chromosome because they are located on unanchored scaffolds in the current genome assembly and were therefore excluded from the chromosomal distribution map. According to their physical positions, the PfNAC genes were sequentially named *PfNAC1–PfNAC108*. Protein sequence analysis revealed that PfNAC proteins varied considerably in length, ranging from 146 amino acids (*PfNAC77*) to 901 amino acids (*PfNAC12*). The predicted molecular weights ranged from 16.23 to 102.28 kDa, and the theoretical isoelectric points (pI) ranged from 4.38 to 9.60, indicating substantial diversity in physicochemical properties among PfNAC proteins (Supplementary Tables S2 and S3).

### 2.2. Multiple Sequence Alignment and Phylogenetic Analysis of PfNACs

**Figure 2:**
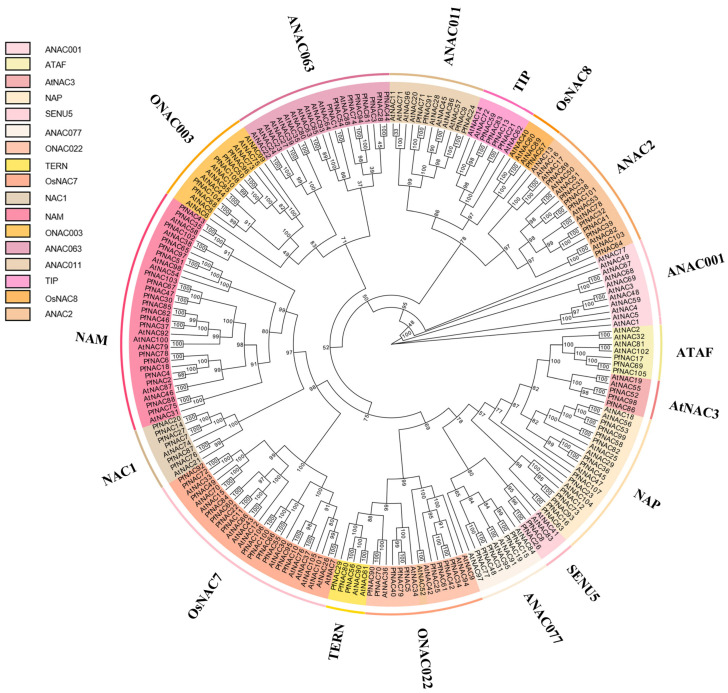
Phylogenetic analysis of NAC proteins from *P. frutescens* and *A. thaliana.* The phylogenetic tree was constructed using the maximum likelihood (ML) method based on the full-length amino acid sequences of 108 PfNAC proteins and 105 AtNAC proteins. Bootstrap values were calculated from 1000 replicates to assess branch support. The NAC proteins were classified into 17 subgroups according to their clustering patterns with the well-characterized NAC proteins from *A. thaliana*. Different subgroups are indicated by different colors.

To explore the structural characteristics of PfNAC proteins, multiple sequence alignment of all 108 PfNAC amino acid sequences was performed. The analysis revealed five conserved subdomains (A–E), which are typical features of the NAC domain (Figure S1). Most PfNAC proteins contained all five conserved subdomains at the N-terminus, indicating a high degree of sequence conservation during evolution. However, several genes, including *PfNAC23*, *PfNAC28*, *PfNAC33*, *PfNAC49*, *PfNAC58*, *PfNAC77*, and *PfNAC82*, exhibited incomplete subdomains. In addition, 31 amino acid residues showed a conservation rate higher than 85%, suggesting strong structural conservation within the PfNAC family. To investigate the evolutionary relationships of NAC proteins, a phylogenetic tree was constructed using the maximum likelihood (ML) method based on the full-length amino acid sequences of 108 PfNAC proteins together with 105 NAC proteins from *Arabidopsis thaliana* (AtNAC) (Figure 2). Because the NAC gene family in *A. thaliana* has been well characterized and classified into distinct subgroups, the PfNAC proteins were classified according to their clustering patterns with AtNAC proteins in the phylogenetic tree. Based on this classification, the PfNAC proteins were divided into 17 subgroups. Among them, the NAM subgroup contained the largest number of members (29), including 21 PfNAC proteins. The OsNAC, ANAC063, and ONAC022 subgroups contained 13, 9, and 9 PfNAC proteins, respectively. In contrast, the OsNAC8 and SENU5 subgroups contained the fewest members (four each), and no PfNAC genes were classified into the ANAC001 subgroup.

### 2.3. Gene Structure and Conserved Motif Analysis of PfNACs

Conserved motif analysis using MEME identified 12 motifs ranging from 11 to 50 amino acids in length (Figure S2). Most PfNAC proteins contained motifs 1–5, which constitute the core DNA-binding region of the NAC domain. The continuous and conserved arrangement of these motifs forms the structural basis for NAC transcription factor functionality. In contrast, a few genes, such as *PfNAC74* and *PfNAC94*, lacked certain core motifs, suggesting potential pseudogenization or functional diversification independent of typical NAC domain activity. Analysis of exon–intron organization revealed that the number of introns ranged from 0 to 8, and exons from 1 to 9 per gene. *PfNAC107* possessed the highest number of exons and introns. Most genes contained 3–4 exons, reflecting structural conservation across the NAC family.

### 2.4. Cis-Acting Regulatory Element Analysis of PfNAC Promoters

**Figure 3:**
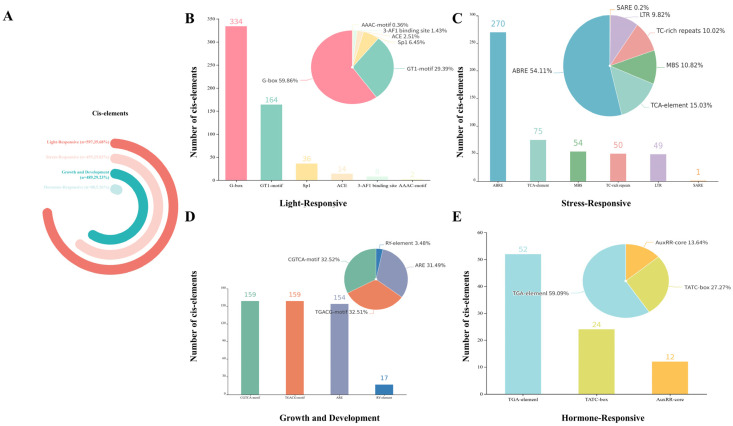
Distribution of cis-acting regulatory elements in the promoter regions of *PfNAC* genes. (**A**) Proportions of the four major categories of cis-acting regulatory elements identified in the 2000 bp upstream regions of *PfNAC* genes. (**B**) Relative abundance of light-responsive elements in the promoters of 108 *PfNAC* genes. (**C**) Relative abundance of stress-responsive elements in the promoters of 108 *PfNAC* genes. (**D**) Relative abundance of growth and development-related elements in the promoters of 108 *PfNAC* genes. (**E**) Relative abundance of hormone-responsive elements in the promoters of 108 *PfNAC* genes.

To investigate the transcriptional regulation of PfNAC genes, cis-acting regulatory elements located within the 2000 bp upstream region of the transcription start site (TSS) were analyzed (Figure 3 and Figure S3; Table S4). A total of 14 stress-related cis-elements were identified and classified into four categories: hormone-responsive, stress-responsive, light-responsive, and development-related elements. Among these categories, light-responsive elements were the most abundant (35.68%), followed by stress-responsive elements (29.83%) and growth and development-related elements (29.23%), hormone-responsive elements were the least represented (5.26%). Notably, G-box, ABRE, CGTCA-motif, TGACG-motif, and ARE were the most frequently detected motifs, with 334, 270, 159, 159, and 154 copies, respectively. All PfNAC genes harbored at least one stress-associated cis-element. Among them, 53 PfNAC genes contained hormone-responsive elements, while 55 genes possessed biotic stress-responsive elements. *PfNAC52* exhibited the highest number of cis-elements (32), followed by *PfNAC98* (31). The high abundance and diversity of cis-regulatory elements suggest that PfNAC genes are extensively involved in plant growth and development, and play crucial roles in mediating responses to biotic and abiotic stresses as well as hormone signaling.

### 2.5. Expression Patterns of PfNAC Genes in Different Tissues of P. frutescens

To explore the tissue-specific expression patterns of PfNAC genes, publicly available RNA-seq datasets from root, stem, and leaf tissues of *P. frutescens* were analyzed. Expression levels were quantified as FPKM, a normalized metric that adjusts for gene length and sequencing depth. For robustness, genes with a mean FPKM < 1 across all samples were filtered out, retaining only those with moderate to high expression for subsequent analysis. A heatmap was generated based on the filtered FPKM values to visualize the expression profiles of PfNAC genes across the three tissues (Figure S4). Hierarchical clustering revealed distinct tissue-specific clusters, with certain genes exhibiting preferential expression in roots, stems, or leaves. Among the 108 PfNAC genes, five showed no detectable expression (FPKM = 0) in any tissue, while 103 were expressed (FPKM ≥ 1) in at least one tissue. Notably, *PfNAC68*, *PfNAC65*, and *PfNAC72* displayed high transcript abundance (FPKM > 10) across all tissues examined, suggesting their potential roles as constitutive regulators.

### 2.6. PfNAC Gene Duplication Events and Synteny Analysis

**Figure 4:**
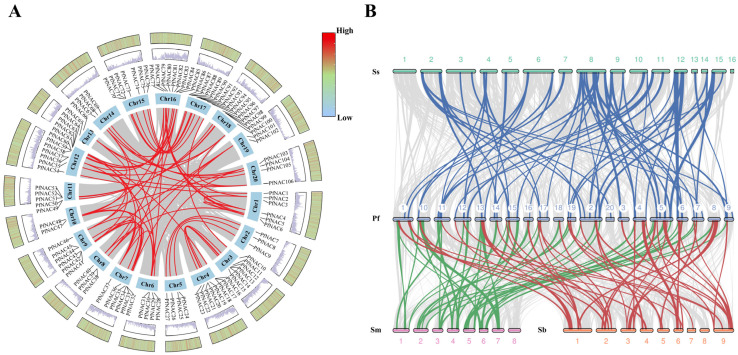
Duplication and synteny analyses of PfNAC genes. (**A**) Analysis of PfNAC gene duplication events. Yellow lines within the circle indicate segmental duplication events; blue and red represent GC content; the heatmap shows the distribution of gene density. (**B**) Synteny analysis between PfNAC transcription factors and those of *S. baicalensis* (Sb), *S. miltiorrhiza* (Sm), and *S. indicum* (Ss). Blue lines indicate syntenic regions between PfNAC and SsNAC genes; green lines indicate syntenic regions between PfNAC and SmNAC genes; red lines indicate syntenic regions between PfNAC and SbNAC genes.

Intraspecific collinearity analysis identified 33 pairs of PfNACs as products of whole-genome duplication (WGD) events (Figure 4). Gene duplication generally contributes to the acquisition of new functions or the maintenance of essential genes that support plant adaptation to changing environments. The nonsynonymous/synonymous substitution ratio (Ka/Ks) for PfNAC pairs ranged from 0.001 to 0.77, all below 1, indicating that the PfNAC gene family underwent strong purifying selection, thereby preserving its functional stability.

To explore evolutionary relationships, interspecific collinearity was analyzed between *P. frutescens* and three related Lamiaceae species—*S. miltiorrhiza*, *Scutellaria baicalensis*, and Sesamum indicum (Figure 4; Supplementary Table S5). A total of 21,245 segmental and 1885 tandem duplication events were identified in *P. frutescens*. Sixty orthologous pairs were found between *P. frutescens* and *S. indicum*, 44 with *S. miltiorrhiza*, and 42 with *S. baicalensis*, suggesting a closer evolutionary relationship with *S. indicum*. PfNACs showing conserved synteny with other species were primarily located on chromosomes 2, 6, 11, 13, and 15, indicating evolutionary conservation, whereas those on chromosome 20 lacked syntenic counterparts, implying lineage-specific diversification.

### 2.7. Subcellular Localization Analysis

**Figure 5:**
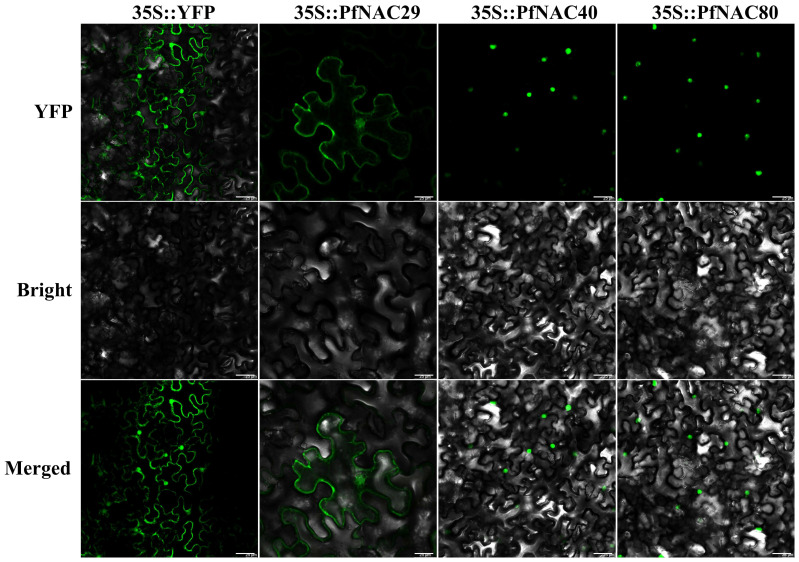
Subcellular localization of *PfNAC29*, *PfNAC40*, and *PfNAC80* proteins in Nicotiana benthamiana leaves (Scale bar = 25 μm).

To determine the subcellular localization of PfNAC proteins, 35S::*PfNAC29/40/80*-PHB-YFP fusion constructs were transiently expressed in Nicotiana benthamiana leaves and observed under confocal microscopy (Figure 5). The empty vector (35S::YFP) showed fluorescence in multiple cellular compartments. Green fluorescence signals from 35S::*PfNAC40*-PHB-YFP and 35S::*PfNAC80*-PHB-YFP constructs were exclusively detected in the nucleus, confirming nuclear localization. In contrast, 35S::*PfNAC29*-PHB-YFP exhibited fluorescence in both the nucleus and the plasma membrane and necleus, indicating dual localization and potential multifunctionality of *PfNAC29*.

### 2.8. Yeast One-Hybrid Assay

**Figure 6:**
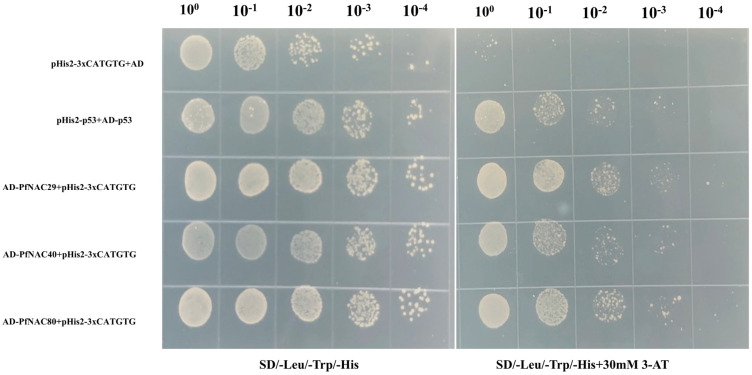
Yeast one-hybrid analysis of the binding of *PfNAC29*, *PfNAC40*, and *PfNAC80* to the CATGTG motif. Y187 yeast cells were grown on SD/-Leu/-Trp/-His plates containing 0 mM 3-aminotriazole (3-AT; control) or 30 mM 3-AT. pHis2-3×CATGTG+AD was used as the negative control, and pHis2-p53+AD-p53 as the positive control.

To verify whether PfNACs can specifically bind to the CATGTG motif, *PfNAC29/40/80*-pGADT7 constructs were co-transformed into yeast with a pHis2-3×CATGTG reporter vector. A concentration of 30 mM 3-amino-1,2,4-triazole (3-AT) was determined to be optimal for selection. As shown in Figure 6, the positive control (pHis2-P53+AD-p53) grew well under both 0 and 30 mM 3-AT conditions, whereas the negative control (pHis2-3×CATGTG+AD) was inhibited at 30 mM 3-AT. Transformants harboring *PfNAC29/40/80*-AD+pHis2-3×CATGTG exhibited growth despite partial inhibition, indicating that all three PfNAC proteins specifically bind to the CATGTG cis-element.

### 2.9. Dual-Luciferase Assay

**Figure 7:**
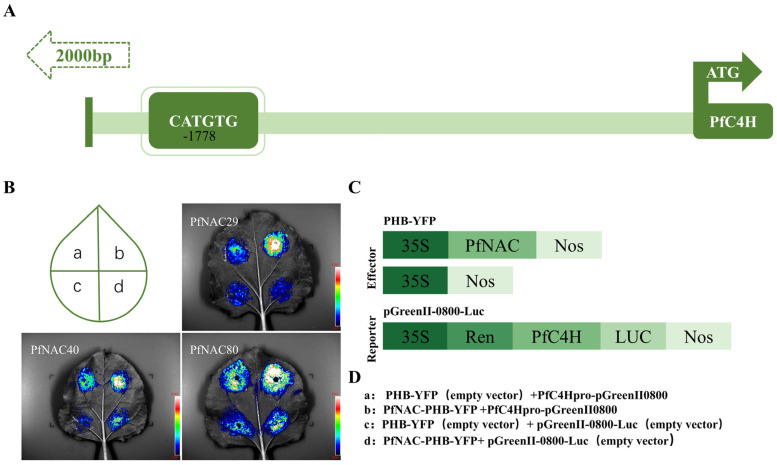
Dual-luciferase assay. (**A**) The 2000 bp promoter region upstream of the *P. frutescens PfC4H* gene. The *PfC4H* promoter contains a CATGTG motif at position 1778 on the reverse strand. (**B**) Results of the transient expression assay in tobacco. (**C**,**D**) Schematic diagrams of the constructs used in this assay.

To explore the transcriptional regulatory activity of PfNACs, dual-luciferase reporter assays were performed using the *PfC4H* promoter as the target. The PfNAC-PHB-YFP effector constructs and pGreenII 0800-LUC reporter plasmids containing the PfC4H promoter were co-infiltrated into *N. benthamiana* leaves (Figure 7). Compared with the empty vector control, co-expression of *PfNAC29*, *PfNAC40*, or *PfNAC80* significantly enhanced luciferase activity, demonstrating that all three transcription factors can activate *PfC4H* promoter-driven expression, thereby positively regulating *PfC4H* transcription.

### 2.10. Overexpression of PfNACs in Transgenic Hairy Roots

**Figure 8:**
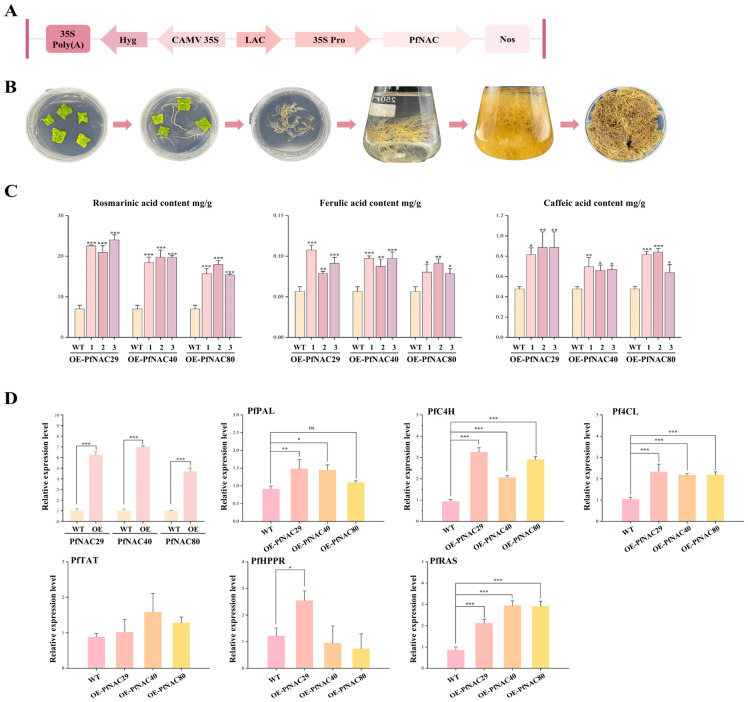
*PfNAC29/40/80* enhance the accumulation of rosmarinic acid (RA), caffeic acid, and ferulic acid. (**A**) Schematic representation of PfNAC overexpression constructs. (**B**) Workflow of transgenic hairy root induction experiments. (**C**) Analysis of phenolic acid contents in PfNAC overexpression lines. (**D**) Expression profiles of key genes involved in phenolic acid biosynthesis. Data are presented as mean ± standard deviation (n = 3), with error bars indicating standard deviation (* *p* < 0.05, ** *p* < 0.01, *** *p* < 0.001, ns, no significant difference.).

To investigate the effect of PfNAC transcription factors on phenolic acid biosynthesis, *PfNAC29/40/80* constructs were introduced into *Agrobacterium rhizogenes* strain K599 and used to transform *P. frutescens* leaf explants. Transgenic hairy roots were induced and verified by PCR using *RolB* and *Hyg* primers (Figure S5). Three independent overexpression lines for each gene were selected for further analysis (Figure 8B). Phenolic acids were quantitatively analyzed by UPLC–MS, and standard curves were generated using OriginPro 2021 (Supplementary Figure S6). The results showed that their levels were significantly higher in the tested roots than in empty vector control (Figure 8C). In *PfNAC29*-overexpressing lines (OE-1/2/3), the contents of rosmarinic acid, caffeic acid, and ferulic acid increased by approximately 3.2-, 1.78-, and 1.23-fold, respectively, compared with the empty-vector control. Similarly, in *PfNAC40*-overexpressing lines, these compounds increased by about 2.70-, 1.20-, and 1.60-fold, whereas in *PfNAC80*-overexpressing lines, the corresponding increases were approximately 2.37-, 1.54-, and 1.40-fold. These results indicate that overexpression of *PfNAC29*, *PfNAC40*, and *PfNAC80* significantly enhances the accumulation of key phenolic acids in *P. frutescens*. Furthermore, qPCR analysis of phenylpropanoid pathway genes (*PfPAL*, *PfC4H*, *Pf4CL*, *PfHPPR*, *PfRAS*, and *PfTAT*) in transgenic and empty vector control revealed distinct expression patterns (Figure 8D). In *PfNAC29*-overexpressing lines, *PfPAL*, *PfC4H*, *Pf4CL*, *PfHPPR*, and *PfRAS* were markedly upregulated, suggesting that *PfNAC29* enhances both phenylalanine and tyrosine metabolism. In *PfNAC40* lines, *PfC4H*, *Pf4CL*, *PfPAL*, and *PfRAS* were upregulated, whereas *PfHPPR* and *PfTAT* remained stable, implying a phenylalanine pathway–dominant regulation. In *PfNAC80* lines, *PfC4H*, *Pf4CL*, and *PfRAS* were significantly induced, supporting a similar regulatory mechanism.

## 3. Discussion

The NAC transcription factor family constitutes a group of plant-specific regulatory proteins that play pivotal roles in diverse biological processes [20]. The number of NAC family members varies substantially among different plant species, reflecting remarkable evolutionary expansion and functional diversification. In this study, 108 *PfNAC* genes were identified in *P. frutescens*, a number comparable to that in *Apium graveolens* (111 members) [21], greater than that in *Lolium perenne* (72 members) [22], but considerably fewer than that in *Brassica napus* (410 members) [23] and *Glycine max* (269 members) [24]. Such differences may be associated with species-specific whole-genome duplication events, gene retention mechanisms, and adaptive evolution, providing key insights into the functional diversification and evolution of the NAC family. Interestingly, the *P. frutescens* genome (1.24 Gb) is much larger than that of *A. thaliana* (125 Mb) and *O. sativa* (466 Mb), yet the number of NAC transcription factors (108 *PfNACs*) is close to that in *A. thaliana* (105) but lower than in *O. sativa* (151) [19,25]. This lack of linear correlation between genome size and NAC gene abundance suggests potential gene loss events during evolution, as also observed in the WRKY and GLK transcription factor families [26,27]. Phylogenetic analysis classified the *PfNACs* into 17 subgroups, consistent with *Eucommia ulmoides* [28], yet differing from *S. miltiorrhiza* (14 subgroups) [29] and *Dactylis glomerata* (14 subgroups) [30], highlighting interspecific diversification. Based on homology with *A. thaliana*, members within the same subgroup likely share related biological functions. For instance, *AtNAC019* (AT1G52890) and *AtNAC055* (AT3G15500) (AtNAC3 subgroup) are induced by drought, salinity, and ABA [31], suggesting similar stress-related roles for *PfNAC52/98/86*. Notably, *PfNAC2*, belonging to the ONAC22 subgroup, was previously shown to positively regulate phenolic acid biosynthesis [12]. Our findings further demonstrate that *PfNAC29* and *PfNAC80*, though belonging to the TERN subgroup, exhibit a convergent regulatory pattern with *PfNAC40* (ONAC22), indicating cross-subgroup functional convergence in activating *PfC4H* transcription. This discovery reveals an evolutionarily conserved yet functionally diversified NAC-mediated regulatory network underlying phenolic acid biosynthesis.

Gene duplication is a fundamental driving force of evolution, contributing to genetic innovation and diversity. In the *PfNAC* family, three pairs of tandemly duplicated genes and 72 pairs of segmentally duplicated genes were identified. Selective pressure analysis revealed that all *PfNAC* gene pairs exhibited Ka/Ks ratios less than 1, indicating strong purifying selection during evolution. This suggests that the *PfNAC* family has maintained functional conservation while limiting deleterious mutations. Analysis of gene structure and conserved motifs provides further insight into the mechanisms underlying transcription factor function and regulation of secondary metabolism. The organization of exons and introns plays a critical role in the evolutionary dynamics of gene families [32]. In this study, most *PfNAC* genes contained two introns and three exons, consistent with findings in *E. ulmoides* [29] and cucumber [33], indicating conserved genetic architecture among NAC genes across species.

Cis-acting elements serve as binding sites for transcription factors and are essential for gene regulation. Promoters, as key transcriptional initiation regions, interact with transcription factors to precisely modulate gene expression, particularly in plant responses to biotic and abiotic stresses. Four major types of cis-elements were identified in the *PfNAC* promoters: hormone-responsive, abiotic stress–responsive, biotic stress and defense–related, and developmental regulation elements. Notably, light-responsive elements were the most abundant (1580 in total), suggesting that *PfNAC* expression may be strongly regulated by light signals. This inference aligns with previous findings that *PfNAC2* promotes phenolic acid accumulation under high-light conditions [12]. Hormonal regulation of NAC genes may also depend on specific cis-elements in their promoters. For example, a gibberellin-responsive element in the *PeNAC1* promoter is essential for its response to gibberellin and salt stress signaling [34]. Similarly, the *PgNAC103* promoter contains MYB-binding sites associated with drought induction, and *PgNAC103* was experimentally validated as a positive regulator of drought stress response [35]. The widespread presence of multiple hormone-related cis-elements in *PfNAC* promoters suggests that this family likely participates in stress adaptation and growth regulation through hormone-mediated signaling pathways.

In *P. frutescens*, *PfNAC2* and *PfGBF3* have been implicated in the regulation of phenolic metabolism under specific conditions, but these studies largely focused on single transcription factors and individual target genes. By contrast, our work provides a systematic view of NAC-mediated regulation by integrating family-wide characterization of PfNAC genes with promoter-binding assays and hairy root overexpression, thereby revealing that *PfNAC29*, *PfNAC40* and *PfNAC80* act as important positive regulators that directly activate *PfC4H* and thereby modulate phenolic acid accumulation. According to phylogenetic analysis, both *PfNAC2* and *PfNAC40* were classified into the ONAC22 subfamily, whereas *PfNAC29* and *PfNAC80* were assigned to the TERN subfamily. *PfNAC29* and *PfNAC80* shared highly conserved motifs, suggesting functional similarity. Although these transcription factors belong to distinct evolutionary branches, functional assays consistently demonstrated that all three—*PfNAC29*, *PfNAC40*, and *PfNAC80*—positively regulate the biosynthesis of phenolic acids in *Perilla frutescens*, indicating a degree of functional redundancy. This phenomenon may be related to the DNA-binding mode of NAC proteins. The NAC domain contains a highly conserved WKATQTD motif that forms an external β-strand, which intercalates into the DNA helix, enabling specific recognition and binding. Structurally, this mode of DNA interaction resembles that of plant-specific WRKY transcription factors, which also employ β-sheet insertion to interact with DNA. Such structural and functional similarities may reflect either a shared ancestral origin between distinct transcription factor families or a convergent evolutionary mechanism driven by similar functional constraints [36]. Alternatively, despite sequence divergence among NAC subfamilies, their core DNA-binding domains may have retained comparable binding specificities [37].

The biosynthesis of plant secondary metabolites is a complex, finely regulated, and multilayered process in which the expression of key enzyme-encoding genes is tightly controlled. Transcription factors act as central “molecular switches” by specifically recognizing and binding to cis-acting elements in the promoter regions of target genes, thereby directly activating or repressing their transcription. In this study, two CATGTG motifs were identified in the promoter region of *PfC4H* in *P. frutescens*. Both yeast one-hybrid and dual-luciferase reporter assays demonstrated that *PfNAC29*, *PfNAC40*, and *PfNAC80* specifically bind to these elements and markedly enhance *PfC4H* transcriptional activity. Overexpression of these transcription factors in transgenic hairy roots significantly promoted the accumulation of rosmarinic acid, caffeic acid, and ferulic acid. Expression profiling revealed that *PfNAC29/40/80* markedly upregulated *PfC4H*, *Pf4CL*, and *PfRAS*, suggesting that they enhance phenolic acid accumulation by promoting the expression of key genes in the phenylalanine-derived pathway, with *PfC4H* being a directly validated target. Notably, *PfNAC29*-overexpressing lines also showed elevated *PfHPPR* expression, implying a potential involvement of *PfNAC29* in both the phenylalanine- and tyrosine-derived branches of phenolic acid biosynthesis, although direct regulation of *PfHPPR* has not been demonstrated. However, this dual-pathway regulation remains speculative, as direct binding of *PfNAC29* to the *PfHPPR* promoter has not yet been experimentally verified. Further yeast one-hybrid (Y1H) or electrophoretic mobility shift assays (EMSA) are required to confirm this hypothesis. Although these analyses were conducted in transgenic hairy roots rather than in whole plants, this system provides a well-established and efficient platform for functional characterization of genes associated with secondary metabolism.

Subcellular localization analysis further revealed distinct functional characteristics among PfNACs. Previous studies have shown that most NAC transcription factors in plants are localized in the nucleus, where they activate downstream target genes involved in maintaining reactive oxygen species (ROS) homeostasis and mediating detoxification under drought stress [38,39,40,41,42]. Consistent with their typical roles as transcriptional regulators, *PfNAC40* and *PfNAC80* were localized in the nucleus. In contrast, some NAC members containing transmembrane motifs have been reported to localize to the plasma membrane and necleus (PM) or endoplasmic reticulum (ER). These membrane-associated NACs can be activated through proteolytic cleavage or phosphorylation, which exposes their nuclear localization signals and allows them to translocate into the nucleus [43]. Notably, *PfNAC29* was localized to both the plasma membrane and the nucleus, suggesting that it may undergo a similar activation process—being released from the membrane and subsequently entering the nucleus to perform transcriptional regulatory functions.

In summary, *PfNAC29*, *PfNAC40*, and *PfNAC80* directly activate *PfC4H* transcription, thereby contributing to enhanced phenolic acid biosynthesis in *P. frutescens* (Figure S8). Despite their homologous relationships, these transcription factors differ in chromosomal distribution, subcellular localization, and evolutionary classification. Notably, *PfNAC29* localizes to both the plasma membrane and nucleus, suggesting a dual regulatory role that links membrane signaling with nuclear transcriptional activation. Phenolic acids, particularly rosmarinic acid, are key antioxidant metabolites that enhance plant tolerance to oxidative and environmental stresses. The *PfNAC29/40/80*-mediated upregulation of phenolic acid biosynthesis thus implies a potential role of these transcription factors in strengthening antioxidant defenses and improving stress resilience in *P. frutescens*. Collectively, these findings provide new insights into the transcriptional diversification of NAC factors and offer valuable molecular tools for metabolic engineering and stress adaptation in medicinal plants.

## 4. Materials and Methods

### 4.1. Identification and Chromosomal Localization of PfNAC Genes

The *P. frutescens* genome dataset (Accession Number: GCA_019511825.1) was obtained from the National Center for Biotechnology Information (NCBI) (https://www.ncbi.nlm.nih.gov) [19]. The hidden Markov model (HMM) profile of the NAC domain (PF02365) was downloaded from the Pfam database (https://pfam.xfam.org/) A genome-wide search was conducted against the *P. frutescens* proteome using HMMER 3.0 with default parameters based on the PF02365 model to identify putative NAC proteins. All candidate *PfNAC* sequences were further validated for the presence of the conserved NAC domain using the SMART database (https://smart.embl.de/, accessed on 12 March 2025) and the NCBI Conserved Domain Database (CDD). Chromosomal mapping of *PfNAC* genes was performed using TBtools v1.120, and their distribution across 20 chromosomes was visualized. Gene names were assigned sequentially according to their physical positions on the chromosomes.

### 4.2. Multiple Sequence Alignment and Phylogenetic Analysis

All identified *PfNAC* protein sequences were exported in FASTA format and aligned using MEGA v11.0 and Jalview v2.11.3.3 to verify conserved residues and compare motif patterns. Sequence logos of conserved motifs were generated with WebLogo [20]; (https://weblogo.threeplusone.com/). Multiple sequence alignments between *P. frutescens* and *A. thaliana* NAC proteins were conducted in MAFFT (https://mafft.cbrc.jp/alignment/software/, accessed on 12 March 2025) under default settings. A maximum likelihood (ML) phylogenetic tree was constructed in IQ-TREE v2.1.2 using the best-fit model JTTDCMut +F +R4, with 1000 bootstrap replicates for branch support. The resulting tree was visualized and annotated in Evolview v3.0.

### 4.3. Gene Structure, Conserved Motif, and Cis-Acting Element Analysis

Conserved motifs in *PfNAC* proteins were identified using MEME Suite v5.5.2 with the following settings: maximum motifs = 15, motif width = 6–50 amino acids, and E-value < 1 × 10^−10^. Gene structures, including exon–intron organization, were analyzed and visualized in TBtools based on the GFF annotation from the *P. frutescens* genome. For promoter analysis, 2000bp upstream sequences from each *PfNAC* translation start site were extracted and submitted to PlantCARE (https://bioinformatics.psb.ugent.be/webtools/plantcare/html/, accessed on 12 March 2025) to predict putative cis-regulatory elements.

### 4.4. Expression Pattern Analysis of PfNACs in Different Tissues

Transcriptomic data corresponding to root, leaf, and stem tissues were downloaded from the *P. frutescens* SRA database at NCBI (https://www.ncbi.nlm.nih.gov/sra/?term=Perilla+frutescens+%28L, accessed on 12 March 2025). Gene expression levels were quantified in FPKM (Fragments Per Kilobase of transcript per Million mapped reads), a normalized measure that accounts for gene length and sequencing depth. FPKM values for candidate PfNAC genes were extracted, and cluster analysis of expression profiles was conducted using TBTools.

### 4.5. Collinearity Analysis of the PfNAC Gene Family

Whole-genome BLASTP (v2.17.0+) analysis was conducted, and the resulting files were formatted for MCScanX (v1.1) analysis (E-value ≤ 1 × 10^−5^). Gene duplication events (tandem, segmental, and orthologous) were classified using MCScanX. Synonymous substitution rates (Ks) were calculated with KaKs_Calculator 2.0, and data with Ks > 2.0 were excluded to avoid saturation. Finally, Circos v0.69-9 was used to visualize the interchromosomal collinearity relationships among *PfNAC* genes.

### 4.6. Plant Materials and Growth Conditions

*P. frutescens* seeds were obtained from the Medicinal Plant Garden of Jilin Agricultural University. The seeds were sown in a mixture of nutrient-enriched soil and vermiculite (3:1, *v*/*v*) and germinated in a growth chamber maintained at 25 °C with a 16 h light/8 h dark photoperiod and 70% relative humidity [44]. Seedlings at the four-leaf stage were used for infection assays. Nicotiana benthamiana plants were cultivated under identical soil conditions and used for subcellular localization assays at the five-pair fully expanded leaf stage.

### 4.7. Subcellular Localization

The coding sequences (CDSs) of *PfNAC29*, *PfNAC40*, and *PfNAC80* were amplified using gene-specific primers containing *HindIII* and *SacI* restriction sites (Table S6). The resulting PCR products were cloned into the PHB-YFP vector under the control of the CaMV 35S promoter to generate the 35S:*PfNAC29/40/80*-PHB-YFP constructs. The recombinant plasmids were introduced into Agrobacterium tumefaciens strain GV3101 (Coolaber, Beijing, China). For infiltration, a single colony was cultured, and the bacterial cells were harvested and resuspended in infiltration buffer (2 mM MgCl_2_, 2 mM MES, 150 μM acetosyringone) to a final OD_600_ of 0.4–0.6. The suspension was then infiltrated into leaves of 30-day-old Nicotiana benthamiana plants [45]. Using a needleless syringe, the bacterial suspension was injected into the abaxial leaf surface until a visible translucent zone (1–2 cm in diameter) was formed; excess suspension was gently wiped away. After a 12 h dark period, the infiltrated plants were returned to standard growth conditions (16 h light/8 h dark photoperiod). YFP fluorescence was observed 48–72 h post-infiltration using a Stellaris 5 confocal laser scanning microscope. YFP signals were captured in the green channel with excitation at 405 nm and emission detection between 420 and 480 nm.

### 4.8. Yeast One-Hybrid (Y1H) Assay

The coding sequences of *PfNAC29, PfNAC40*, and *PfNAC80* were amplified and cloned into the pGADT7 vector using the *EcoRI* and *BamHI* restriction sites to generate the constructs pGADT7-*PfNAC29/40/80*. Three tandem repeats of the CATGTG fragment were synthesized and inserted into the pHIS2 vector at the *SacI* and *EcoRI* sites to obtain the reporter construct pHIS2-3xCATGTG). The recombinant plasmids were co-transformed into Saccharomyces cerevisiae strain Y187 (Coolaber, Beijing, China). Transformed yeast cells were cultured at 29 °C with shaking (180 rpm) until reaching an optical density of approximately OD_600_ ≈ 1.0. The cultures were then serially diluted and spotted onto SD/-Trp/-Leu/-His medium supplemented with different concentrations of 3-amino-1,2,4-triazole (3-AT) to determine the optimal selective concentration. After optimization, yeast cells carrying the pGADT7-*PfNAC29/40/80* constructs were spotted onto SD/-Trp/-Leu/-His plates containing 0 or 30 mM 3-AT. Growth differences were used to evaluate the protein–DNA interactions. The pHIS2-AD combination served as a negative control, while the pGADT7-p53 + pHIS2-rec-p53 pair was used as a positive control.

### 4.9. Dual-Luciferase Reporter Assay

The 2000 bp promoter region upstream of *PfC4H* was analyzed using PlantCARE to identify CATGTG cis-elements. The *PfC4H* promoter fragment (*PfC4H-pro*) was amplified and cloned into the pGreenII0800-LUC vector using *SalI* and *HindIII* sites. The coding sequences of *PfNAC29*, *PfNAC40*, and *PfNAC80* were inserted into the PHB-YFP vector under the CaMV 35S promoter. All recombinant plasmids were introduced into *A. tumefaciens* strain GV3101 (pSoup-P19). Transient expression was performed in *N. benthamiana* leaves (~30 days old) as described above. After 24 h of dark incubation, plants were grown under normal light/dark conditions for 48–72 h. Luciferase substrate Coolaber (Beijing, China) (0.3 mg/mL) was sprayed evenly on the abaxial leaf surface, incubated in darkness for 10 min, and luminescence was detected using a plant imaging system.

### 4.10. Vector Construction and Genetic Transformation

The full-length CDS of *PfNAC29*, *PfNAC40*, and *PfNAC80* were inserted into the pCAMBIA1304 plant overexpression vector under the control of the CaMV 35S promoter using *BglII* and *BstEII* restriction sites. The resulting constructs were transferred into *A. rhizogenes* strain K599 and used for hairy root induction in *P. frutescens* leaf explants via the leaf-disk infection method. Leaf explants were first washed under running tap water for 10 min, surface-sterilized with 70% ethanol for 30 s, followed by 1.0% (*w*/*v*) sodium hypochlorite solution for 8–10 min, and then rinsed three times with sterile distilled water before inoculation. *A. rhizogenes* cultures harboring recombinant plasmids were activated in YEB medium containing 50 mg/L kanamycin and 50 mg/L streptomycin at 28 °C with shaking at 180 rpm for 3–4 subcultures. Bacterial suspensions were adjusted to OD_600_ = 0.6–0.8 using half-strength MS liquid medium and used to inoculate the sterilized explants by shaking at 28 °C, 180 rpm for 15 min. After blotting dry, explants were co-cultivated on antibiotic-free 1/2 MS solid medium for 2–3 days, then washed with sterile water and transferred to 1/2 MS medium containing 500 mg/L cefotaxime for bacterial elimination. The cefotaxime concentration was gradually reduced at 7-day intervals until no bacterial growth was observed. Hairy roots were subsequently subcultured in 1/2 MS liquid medium. Genomic DNA was extracted from hairy roots, and PCR amplification using *rolB* and *hygromycin* (*Hyg*) primers was performed to identify positive transgenic lines for subsequent analysis.

### 4.11. qRT-PCR Analysis

Total RNA was extracted from *Perilla frutescens* hairy roots using the YALEPIC^®^ Plant Total RNA Rapid Extraction Kit (TransGen, Beijing, China). First-strand cDNA was synthesized from purified RNA with the TransScript^®^ Uni All-in-One First-Strand cDNA Synthesis SuperMix for qPCR (with One-Step gDNA Removal) (TRAN, Beijing, China), following the manufacturer’s protocol. Quantitative real-time PCR (qRT-PCR) was conducted on a Roche LightCycler 96 system using SYBR Green I dye. Relative transcript levels were calculated using the 2^−ΔΔCT^ method, with *PfActin* (GenBank accession no. AB002819.1) as the internal control. Primer sequences used for qRT-PCR are listed in Supplementary Table S6.

### 4.12. UPLC–MS Analysis of Phenolic Acids in Transgenic Hairy Roots

Standard solutions of rosmarinic acid, ferulic acid, and caffeic acid were prepared in methanol at concentrations of 0.1196, 0.0827, and 0.096 mg/mL, respectively. Freeze-dried hairy roots (20 mg per line) were ground into powder and extracted with 1 mL methanol by sonication for 1 h. The extracts were filtered through 0.22 µm membranes prior to analysis. UPLC–MS analysis was performed with a mobile phase consisting of 0.1% formic acid in acetonitrile (A) and water (B) under the following gradient conditions: 0–7 min, 5–20% A; 7–11 min, 20–22% A; 11–20 min, 22–60% A; 20–25 min, 60–65% A; 25–28 min, 65% A; 28–30 min, 65–95% A; and 30–34 min, 95% A. The flow rate was maintained at 0.3 mL/min, the column temperature at 35 °C, and the injection volume at 2 µL. Mass spectrometry was conducted in negative ion mode using a heated electrospray ionization (HESI) source at 3.0 kV. The full MS scan range was *m*/*z* 100–1500, with a resolution of 70,000 for full MS and 17,500 for MS/MS. The sheath gas and auxiliary gas flow rates were 35 and 10 arb, respectively. The ion transfer tube temperature was 320 °C, the auxiliary gas temperature was 310 °C, and the collision energy was set at 35 eV.

### 4.13. Statistical Analysis

Statistical analyses were performed using IBM SPSS (V31) Statistics (IBM Corp., Armonk, NY, USA). For comparisons between two groups, a two-tailed Student’s *t*-test was applied. For comparisons among more than two groups, one-way analysis of variance (one-way ANOVA) followed by Duncan’s post hoc multiple comparison test was used. Differences were considered statistically significant at *p* < 0.05 (* *p* < 0.05; ** *p* < 0.01; *** *p* < 0.001). All measurements were obtained from three independent biological replicates, and data are expressed as mean ± standard deviation (mean ± SD).

## 5. Conclusions

In this study, we systematically identified and characterized the PfNAC gene family in *P. frutescens*, revealing 108 members distributed across 20 chromosomes and grouped into 17 subfamilies. Among them, PfNAC29, PfNAC40, and PfNAC80 were identified as key regulators potentially involved in phenolic acid biosynthesis. Subcellular localization analysis showed that PfNAC40 and PfNAC80 were confined to the nucleus, whereas PfNAC29 was localized in both the plasma membrane and nucleus, indicating a dual regulatory role that may integrate membrane signaling with nuclear transcription. Yeast one-hybrid assays confirmed that all three proteins specifically recognize and bind to the CATGTG cis-element in the PfC4H promoter. Overexpression of these genes in hairy roots significantly enhanced the accumulation of phenolic acids by upregulating biosynthetic enzyme genes. Phenolic acids, particularly rosmarinic acid, are known antioxidant metabolites that mitigate oxidative damage and enhance plant tolerance to environmental stresses. Therefore, the PfNAC29/40/80-mediated activation of phenolic acid biosynthesis may contribute to improved antioxidant defense and stress resilience in *P. frutescens*. Collectively, these findings provide new insights into the transcriptional diversification of NAC factors and highlight their potential in metabolic engineering and adaptive stress regulation of medicinal plants.

## Data Availability

Data are contained within the article and Supplementary Materials.

## Supplementary Materials

The following supporting information can be downloaded at: https://www.mdpi.com/article/10.3390/plants15060922/s1. Reference [46] is cited in the Supplementary Materials.
