# Supplementary material for: Identification and Validation of NAC Transcription Factors Enhancing Phenolic Acid Production in Perilla frutescens
Source: Plants (Basel). 2026 Mar 17;15(6):922. doi: 10.3390/plants15060922 (PMC13030785; doi:10.3390/plants15060922)
Supplement: Supplementary file 1 [file plants-15-00922-s001.zip › Supplementary Figures.pdf]

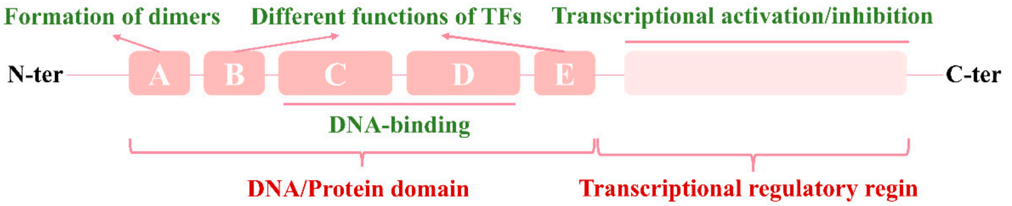

Figure S1. Schematic representation of the PfNAC protein domain structures.

The NAC transcription factors contain a conserved N-terminal NAC domain (DNA-binding) and a variable C-terminal transcriptional regulatory region. The diagram was drawn based on the conserved domain analysis of PfNAC proteins.

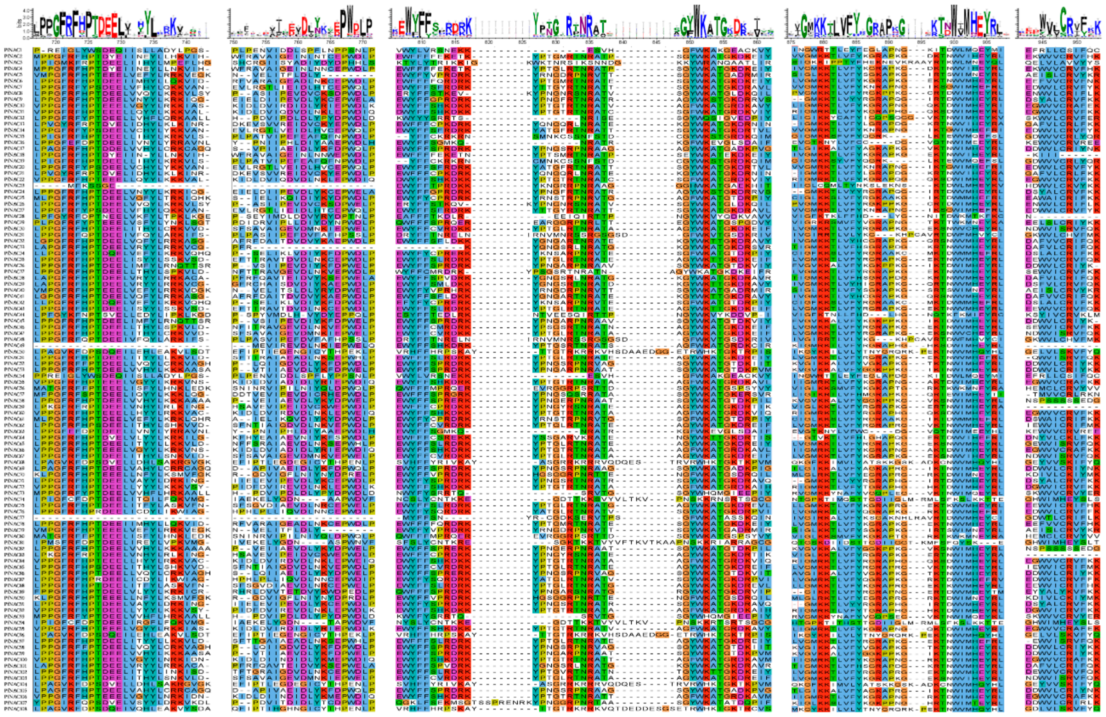

Figure S2. Multiple sequence alignment and structural characterization of PfNAC proteins.

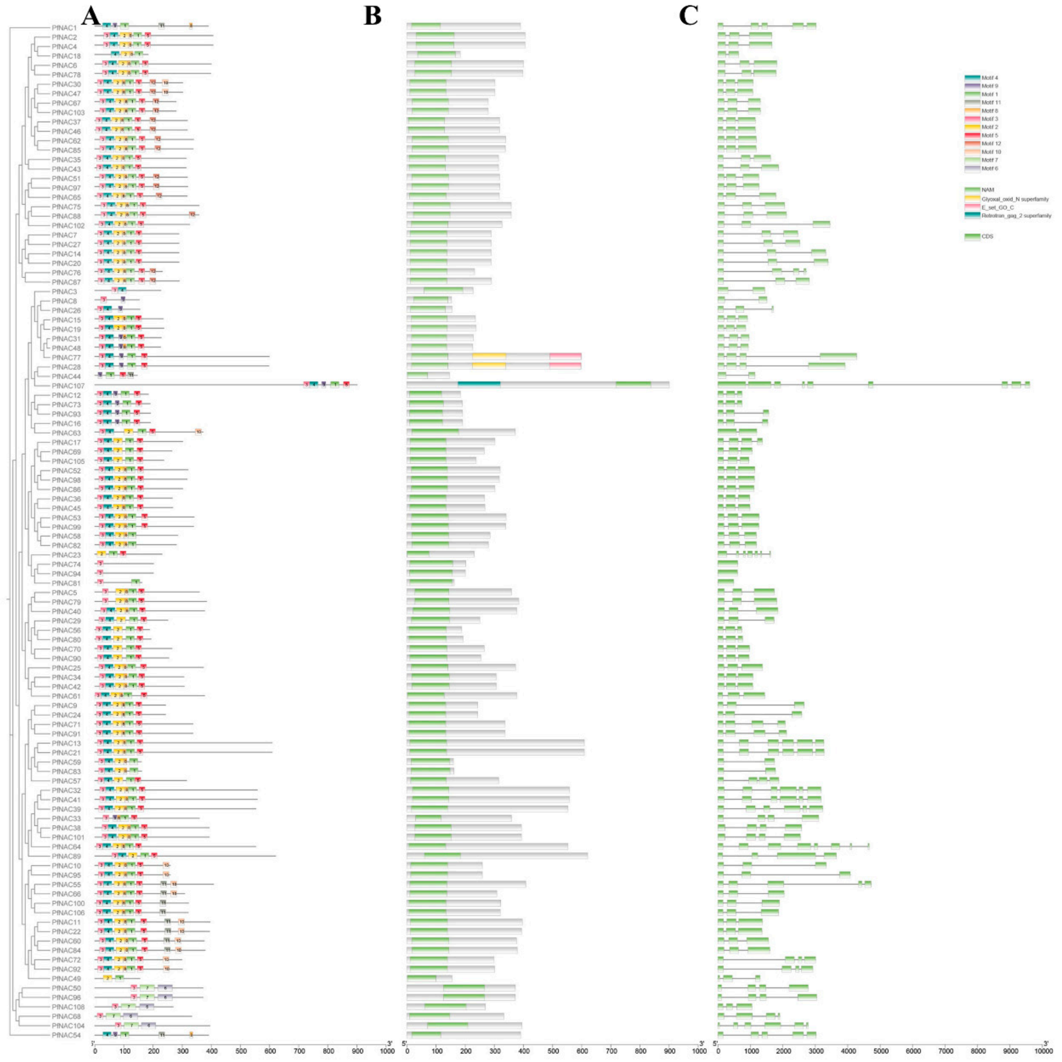

Figure S3. Analysis of conserved motifs and gene structures of PfNAC proteins.

(A) Distribution of conserved motifs of PfNACs, with motifs indicated by different colored boxes and gray lines indicating relative lengths of proteins. (B) Conserved domains of PfNAC proteins, indicated by different colored rectangle. (C) Exon/intron structure of PfNAC proteins. The exons and introns are represented by green boxes and black lines, respectively.

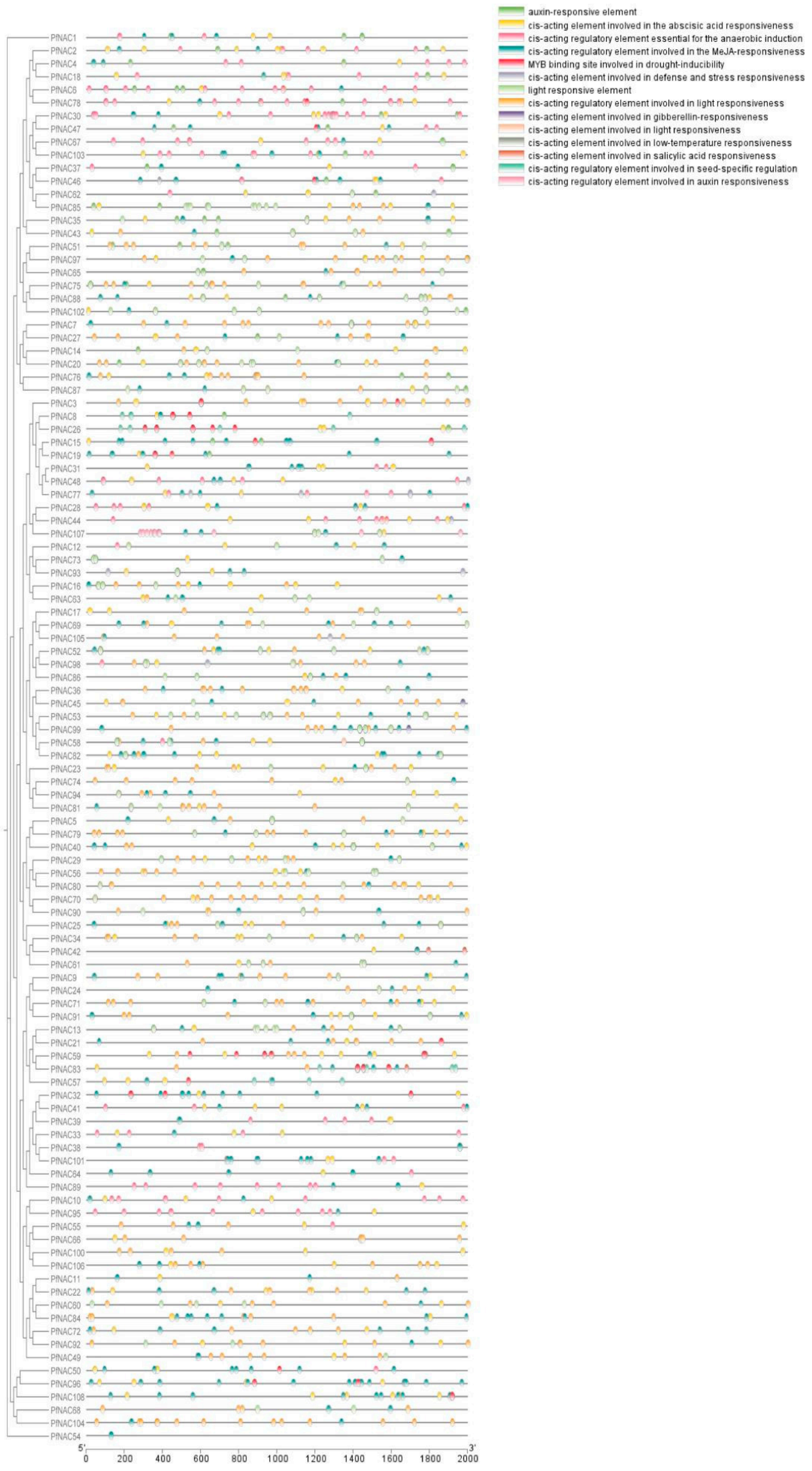
auxin-responsive element
cis-acting element involved in the abscisic acid responsiveness
cis-acting regulatory element essential for the anaerobic induction
cis-acting regulatory element involved in the MeJA-responsiveness
MYB binding site involved in drought-inducibility
cis-acting element involved in defense and stress responsiveness
light responsive element
cis-acting regulatory element involved in light responsiveness
cis-acting element involved in gibberellin-responsiveness
cis-acting element involved in light responsiveness
cis-acting element involved in low-temperature responsiveness
cis-acting element involved in salicylic acid responsiveness
cis-acting regulatory element involved in seed-specific regulation
cis-acting regulatory element involved in auxin responsiveness
5'
3'
0
200
400
600
800
1000
1200
1400
1600
1800
2000

Figure S4. Prediction of cis-acting elements in PfNAC gene promoters.

The 2000 bp upstream sequences of PfNAC genes were analyzed to identify putative cis-acting regulatory elements using the PlantCARE database. The identified elements were categorized into different functional groups, including hormone-responsive elements, light-responsive elements, stress-related elements, and growth and development-related elements. Different colors represent different types of cis-acting elements.

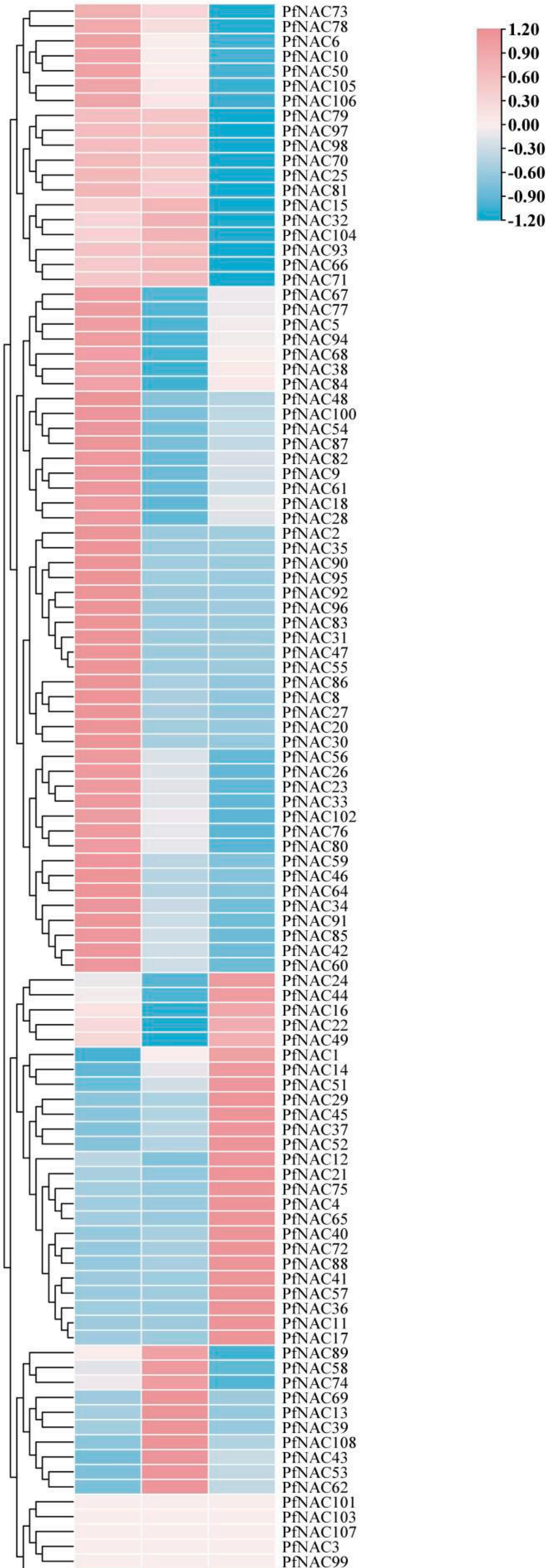
PfNAC73
PfNAC78
PfNAC6
PfNAC10
PfNAC50
PfNAC105
PfNAC106
PfNAC79
PfNAC97
PfNAC98
PfNAC70
PfNAC25
PfNAC81
PfNAC15
PfNAC32
PfNAC104
PfNAC93
PfNAC66
PfNAC71
PfNAC67
PfNAC77
PfNAC5
PfNAC94
PfNAC68
PfNAC38
PfNAC84
PfNAC48
PfNAC100
PfNAC54
PfNAC87
PfNAC82
PfNAC9
PfNAC61
PfNAC18
PfNAC28
PfNAC2
PfNAC35
PfNAC90
PfNAC95
PfNAC92
PfNAC96
PfNAC83
PfNAC31
PfNAC47
PfNAC55
PfNAC86
PfNAC8
PfNAC27
PfNAC20
PfNAC30
PfNAC56
PfNAC26
PfNAC23
PfNAC33
PfNAC102
PfNAC76
PfNAC80
PfNAC59
PfNAC46
PfNAC64
PfNAC34
PfNAC91
PfNAC85
PfNAC42
PfNAC60
PfNAC24
PfNAC44
PfNAC16
PfNAC22
PfNAC49
PfNAC1
PfNAC14
PfNAC51
PfNAC29
PfNAC45
PfNAC37
PfNAC52
PfNAC12
PfNAC21
PfNAC75
PfNAC4
PfNAC65
PfNAC40
PfNAC72
PfNAC88
PfNAC41
PfNAC57
PfNAC36
PfNAC11
PfNAC17
PfNAC89
PfNAC58
PfNAC74
PfNAC69
PfNAC13
PfNAC39
PfNAC108
PfNAC43
PfNAC53
PfNAC62
PfNAC101
PfNAC103
PfNAC107
PfNAC3
PfNAC99
PfNAC19
PfNAC7
PfNAC63
R
S
L
1.20
0.90
0.60
0.30
0.00
-0.30
-0.60
-0.90
-1.20

Figure S5. Relative expression level of the *PfNACs* gene family in the roots, stems and leaves of *P.frutescens*.

The color scale at the right of the heatmap refers to the relative expression level, and the color gradient from blue to pink represents increasing expression level. [1]

1. Ming, R.; Fang, T.; Ling, W.; Geng, J.; Qu, J.; Zhang, Y.; Chen, J.; Yao, S.; Li, L.; Huang, D.; et al. The GRAS Transcription Factor PtrPAT1 of Poncirus Trifoliata Functions in Cold Tolerance and Modulates Glycine Betaine Content by Regulating the BADH-like Gene. *Hortic. Res.* **2025**, *12*, uhae296, doi:10.1093/hr/uhae296.

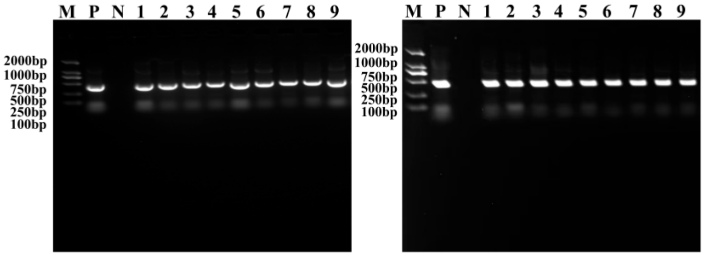

Figure S6. PCR identification of transgenic hairy roots.Lane M: DNA marker (DL2000); P: positive plasmid control; N: negative control.(A) PCR amplification results of the rolB gene in PfNAC29/40/80 transgenic lines.(B) PCR amplification results of the Hyg gene in PfNAC29/40/80 transgenic lines.

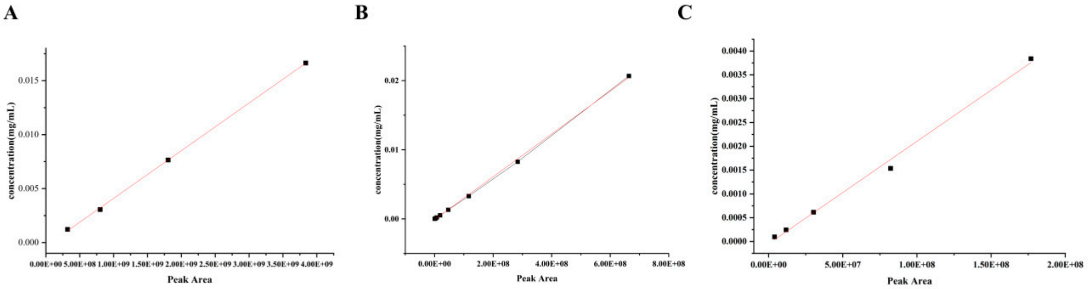

Figure S7. Standard curves for phenolic acid quantification. (A) Standard curve for rosmarinic acid (RA) ($y = a + bx$; $a = -3.16523 \times 10^{-4}$, $b = 4.41454 \times 10^{-11}$, $R^2 = 0.99956$). (B) Standard curve for ferulic acid ($y = a + bx$; $a = -1.01962 \times 10^{-4}$, $b = 3.09754 \times 10^{-11}$, $R^2 = 0.99920$). (C) Standard curve for caffeic acid ($y = a + bx$; $a = -4.52151 \times 10^{-5}$, $b = 2.15088 \times 10^{-11}$, $R^2 = 0.99351$).

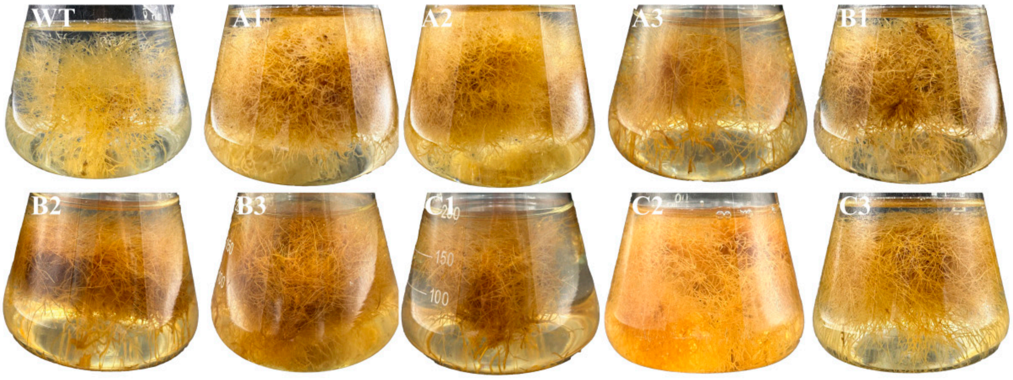

Figure S8. Growth phenotypes of transgenic hairy roots. WT, wild-type control line. A1–A3, PfNAC29 transgenic lines; B1–B3, PfNAC40 transgenic lines; C1–C3, PfNAC80 transgenic lines.

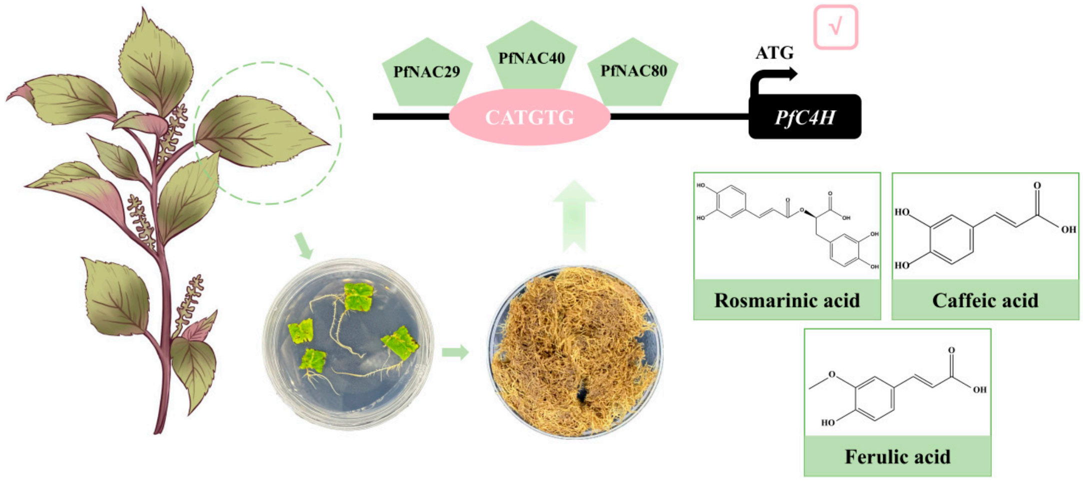

Figure S9. Model of *PfNAC29/40/80*-mediated regulation of phenolic acid biosynthesis in *P.frutescens*. *PfNAC29, PfNAC40* and *PfNAC80* activate *PfC4H* transcription by binding to the CATGTG motif in its promoter, thereby promoting the accumulation of rosmarinic acid, caffeic acid and ferulic acid.
